# Methyl Farnesoate Plays a Dual Role in Regulating *Drosophila* Metamorphosis

**DOI:** 10.1371/journal.pgen.1005038

**Published:** 2015-03-16

**Authors:** Di Wen, Crisalejandra Rivera-Perez, Mohamed Abdou, Qiangqiang Jia, Qianyu He, Xi Liu, Ola Zyaan, Jingjing Xu, William G. Bendena, Stephen S. Tobe, Fernando G. Noriega, Subba R. Palli, Jian Wang, Sheng Li

**Affiliations:** 1 Key Laboratory of Insect Developmental and Evolutionary Biology, Institute of Plant Physiology and Ecology, Shanghai Institutes for Biological Sciences, Chinese Academy of Sciences, Shanghai, China; 2 Department of Life Science, Qiannan Normal College for Nationalities, Duyun, Guizhou, China; 3 Department of Biological Sciences, Florida International University, Miami, Florida, United States of America; 4 Department of Entomology, University of Maryland, College Park, Maryland, United States of America; 5 Department of Entomology, College of Agriculture, University of Kentucky, Lexington, Kentucky, United States of America; 6 Department of Biology, Queen’s University, Kingston, Ontario, Canada; 7 Department of Cell and Systems Biology, University of Toronto, Toronto, Ontario, Canada; K.U.Leuven, BELGIUM

## Abstract

*Corpus allatum* (CA) ablation results in juvenile hormone (JH) deficiency and pupal lethality in *Drosophila*. The fly CA produces and releases three sesquiterpenoid hormones: JH III bisepoxide (JHB3), JH III, and methyl farnesoate (MF). In the whole body extracts, MF is the most abundant sesquiterpenoid, followed by JHB3 and JH III. Knockout of JH acid methyl transferase (*jhamt*) did not result in lethality; it decreased biosynthesis of JHB3, but MF biosynthesis was not affected. RNAi-mediated reduction of 3-hydroxy-3-methylglutaryl CoA reductase (*hmgcr*) expression in the CA decreased biosynthesis and titers of the three sesquiterpenoids, resulting in partial lethality. Reducing *hmgcr* expression in the CA of the *jhamt* mutant further decreased MF titer to a very low level, and caused complete lethality. JH III, JHB3, and MF function through Met and Gce, the two JH receptors, and induce expression of *Kr-h1*, a JH primary-response gene. As well, a portion of MF is converted to JHB3 in the hemolymph or peripheral tissues. Topical application of JHB3, JH III, or MF precluded lethality in JH-deficient animals, but not in the *Met gce* double mutant. Taken together, these experiments show that MF is produced by the larval CA and released into the hemolymph, from where it exerts its anti-metamorphic effects indirectly after conversion to JHB3, as well as acting as a hormone itself through the two JH receptors, Met and Gce.

## Author Summary

Methyl farnesoate (MF) is the immediate precursor of juvenile hormone (JH) III in the JH biosynthetic pathway, and lacks the epoxide moiety characteristic of JHs. The potential role of MF as a JH in arthropods has been an issue of a long-standing debate. In this report, comprehensive molecular genetics studies demonstrated that MF plays a dual role in regulating *Drosophila* metamorphosis. MF is produced by the larval CA and released into the hemolymph, from where it exerted its anti-metamorphic effects indirectly after conversion to JHB3, as well as acting as a hormone itself through a direct interaction with Met and Gce, the two JH receptors.

## Introduction

Juvenile hormones (JHs) are members of a family of sesquiterpenoid compounds synthesized primarily by the *corpus allatum* (CA) of insects. Several forms of JH have been identified, including JH 0, JH I, 4-methyl JH I, JH II, JH III, JH bisepoxide (JHB3) and JH skipped bisepoxide. JH III is found in most insect orders, whereas JH 0, JH I, and JH II are exclusive to Lepidoptera [1]. JHB3 is unique to higher Diptera, such as the fruit fly, *Drosophila melanogaster* [2], and JH skipped bisepoxide has been described in Heteroptera [3]. Methyl farnesoate (MF) is the major sesquiterpenoid identified in the hemolymph of crustaceans, in which it might play the role of a JH [4]. MF lacks the epoxide moiety present in other JHs, and it is usually considered as an immediate precursor of JH III in Insecta [1]. The potential role of MF as a true JH in insects has been an issue of a long-standing debate; it has JH activity in the *Drosophila* white puparial bioassays and is abundant in the hemolymph of several insects [5–10].

The biosynthetic pathway of JH III in the CA of insects involves 13 discrete enzymatic reactions and is conventionally divided into early and late steps (S1 Fig) [1]. The early steps follow the mevalonic acid pathway to form farnesyl pyrophosphate [11]. 3-hydroxy-3-methylglutaryl CoA reductase (HMGCR), the rate-limiting enzyme for mevalonic acid biosynthesis in mammals, is also an important enzyme in the early steps of JH biosynthesis [11]. In the late steps of JH III biosynthesis, farnesyl pyrophosphate is sequentially transformed to farnesol, farnesal and farnesoic acid (FA) [1]. The order of the last two biosynthetic steps, methyl esterification and epoxidation, catalyzed by a JH acid (JHA) methyltransferase (JHAMT) and a P450 epoxidase, differs among insect species: epoxidation precedes methylation in Lepidoptera, whereas epoxidation follows methylation in Diptera, Orthoptera, Dictyoptera, Coleoptera and probably most other insect orders [12–17]. The *Drosophila* CA produces and releases three sesquiterpenoids: JHB3, JH III, and MF [2, 9, 10, 18, 19]. However, the entire JH biosynthetic pathway in *Drosophila* has not been well defined to date.

One major function of JH is to inhibit action of the molting hormone (20-hydroxyecdysone, 20E) for preventing metamorphosis during the larval molts [1]. In JH-deficient animals in which the CA is genetically ablated, JH prevents 20E-triggered apoptosis of the larval fat body [20, 21] and precocious differentiation of the optic lobe in the adult brain [22] in *Drosophila*. JH serves an equally important function, regulating various aspects of reproductive maturation in most insects [1]. For example, incomplete ablation of the CA results in a partial deficiency of JH with an associated reduction in reproductive capacity in *Drosophila* [23].

The recent discovery that the JH-resistance gene, *Methoprene-tolerant* (*Met*), plays a critical role in insect metamorphosis has been followed by a rapid increase in our understanding of JH signaling [24]. Met and Gce, two paralogous bHLH transcription factors in *Drosophila*, are involved in JH action [25, 26]. Although both the *Met* and *gce* null mutants are viable, the *Met gce* double mutant dies during the larval-pupal transition [21], similar to that observed in JH-deficient animals [20, 22]. Functionally, Met and Gce mediate JH action to prevent the 20E-triggered metamorphic events [20–22]. Moreover, Met and Gce bind to JH at physiological concentrations *in vitro* [27, 28], suggesting that they are JH receptors. In parallel, Met is also involved in JH action as a receptor in the red flour beetle, *Tribolium castaneum* [28, 29]. Downstream of Met, the anti-metamorphic action of JH is transduced by Krüppel-homolog 1 (Kr-h1), a transcription factor involved in JH action. A number of studies in *Drosophila* [21, 30, 31] and several other insect species [24] have shown that *Kr-h1* is a JH primary-response gene.

As shown in previous studies [20, 22], genetic ablation of the CA results in JH deficiency and pupal lethality in *Drosophila*. To further clarify the roles of JHs in *Drosophila*, we generated a *jhamt* mutant. Surprisingly, the *jhamt* mutant is viable and its MF biosynthesis was not affected. Further, MF was demonstrated to exert crucial roles for completion of *Drosophila* metamorphosis, by both acting directly as a JH and indirectly after conversion to JHB3.

## Results

### Mutation of *jhamt* did not increase JH-dependent lethality

**Fig 1:**
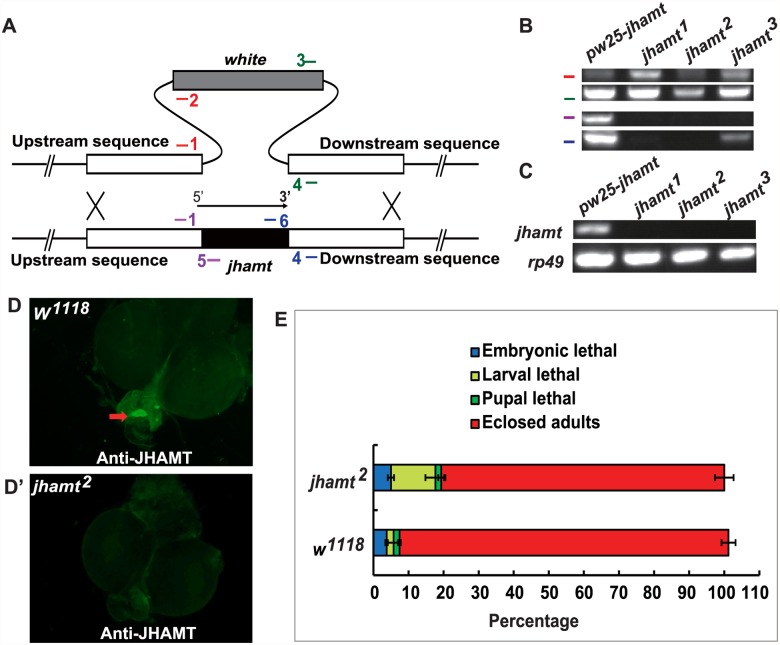
Generation and characterization of the *jhamt* mutants. (A) Scheme for *jhamt* targeting. *jhamt* (black box) is replaced with *white* (gray box) by homologous recombination of the flanking sequences (white boxes). Red bars represent the primer pairs *jhamt-1/jhamt-2*; green bars, *jhamt-3/jhamt-4*; purple bars, *jhamt-1/jhamt-5*; blue bars, *jhamt-6/jhamt-4*. (B) Genomic DNA PCR to detect *white* and *jhamt* DNA using the above-mentioned primer pairs and genomic DNA extracted from *pw25-jhamt* and *jhamt* ^*1*^, *jhamt* ^*2*^, and *jhamt* ^*3*^ lines. (C) Reverse transcription PCR to detect *jhamt* mRNA (*rp49* as the internal control) from *pw25-jhamt* and *jhamt* ^*1*^, *jhamt* ^*2*^, and *jhamt* ^*3*^. All of the mRNAs were isolated from the brain-RG complexes at 3 hours after initiation of wandering (3hAIW). (D and D’) Immunohistochemistry to detect JHAMT in the CA of *w* ^*1118*^ (D) and *jhamt* ^*2*^ (D’) at 3hAIW. The red arrow points to the CA showing JHAMT expression. (E) The lethality of *w* ^*1118*^ and *jhamt* ^*2*^ homozygous mutant during embryonic, larval and pupal stages.

Genetic ablation of the CA results in JH deficiency and pupal lethality in *Drosophila* [20, 22], while traces of the CA cells are often still present in the ring gland (RG) of the ablated animals during the early larval stages. To further clarify the roles of JHB3, JH III, and MF in *Drosophila*, we generated a *jhamt* mutant, which was expected to disrupt the JH biosynthetic pathway and to result in lethality at pupal or earlier stages. The ends-out gene targeting method was utilized to replace the entire *jhamt* open reading frame with the *white* gene via homologous recombination [32] (Fig. 1A). Three independent *jhamt* mutant lines (*jhamt* ^*1*^, *jhamt* ^*2*^, and *jhamt* ^*3*^) were obtained and validated by PCR analysis of genomic DNA (Fig. 1B). The mRNA of *jhamt* was not detectable in the CA of the *jhamt* mutants at 3 h after the initiation of wandering (3h AIW), a time when JH titer [10], JH biosynthesis [2] and *jhamt* mRNA levels [13] are high (Fig. 1C). Immunohistochemical studies revealed the absence of JHAMT protein in the CA of the *jhamt* mutants at 3h AIW (Fig. 1D and 1D’). Taken together, these studies showed that *jhamt* ^*1*^ and *jhamt* ^2^ are null alleles. For consistence, *jhamt* ^*2*^ was used in all the subsequent studies.

JH-dependent phenotypes were evaluated in *jhamt* ^*2*^ in comparison with *w* ^*1118*^, the wild type fly used to generate *jhamt* mutants. Approximately 10% of *jhamt* ^*2*^ larvae died during the larval stage, with the rest surviving to adulthood (Fig. 1E). In addition, the initiation of wandering was delayed for about 4 hours in *jhamt* ^*2*^ larvae (S2A Fig), whereas body weight was not affected (S2B Fig). The fecundity of *jhamt* ^*2*^ adult females decreased by about 80%, whereas topical application of methoprene (0.5×10^-3^ μmol per female) partially restored fecundity (S2C Fig). The ovary size of the 6-day-old *jhamt* ^*2*^ virgin females was significantly reduced. However, methoprene partially restored ovary growth (S2C’ Fig). The CA-specific *Aug21-GAL4* was used for genetic ablation of the CA in previous studies [20, 22]. We performed a genetic rescue experiment with *Aug21-GAL4* driving *UAS-jhamt* overexpression in a *jhamt* ^*2*^ background. Importantly, fecundity and ovary growth of *jhamt* ^*2*^ */jhamt* ^*2*^; *Aug21-GAL4*>*UAS-jhamt* were restored to similar levels to those in *w* ^*1118*^ (S2D and S2D’ Fig), showing that the reproductive capacity in *jhamt* ^*2*^ was fully rescued by CA-specific *jhamt* overexpression. Overall, the phenotypic changes in *jhamt* ^*2*^ were similar to those described for *Aug21-GAL4*>*UAS-reaper*::*UAS-hid* animals, in which the CA is incompletely ablated and JH is partially deficient [23]. However, *jhamt* ^*2*^ showed less robust effects than those observed in JH-deficient *Aug21-GAL4*>*UAS-Grim* (*Aug21*>*Grim*) animals, in which CA activity is efficiently disrupted [20, 22].

### Mutation of *jhamt* decreased JHB3 but not MF biosynthesis

**Fig 2:**
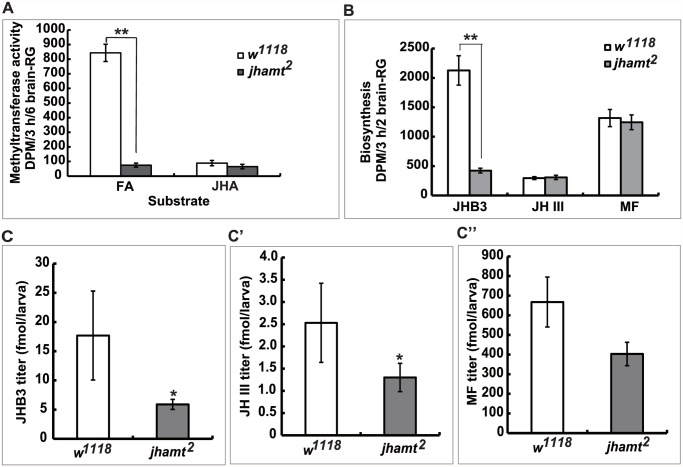
Mutation of *jhamt* decreases JHB3 but not JH III and MF biosynthesis. (A) Measurements of methyltransferase activity in the brain-RG complexes in *w* ^*1118*^ and *jhamt* ^*2*^ at 3hAIW using FA or JHA as the substrate. (B) Measurements of JH biosynthesis in the brain-RG complexes in *w* ^*1118*^ and *jhamt* ^*2*^ at 3hAIW using the RCA-TLC method. (C-C”) Quantitative measurements of whole body titers of JHB3 (C), JH III (C’), and MF (C”) in *w* ^*1118*^ and *jhamt* ^*2*^ at 3hAIW using the HPLC-FD protocol.

To verify whether *jhamt* ^*2*^ might be only partially JH-deficient, we measured the activity of methyltransferase in the brain-RG complexes isolated from 3h AIW larvae using either FA or JHA as substrates [14, 20, 23, 33]. In *w* ^*1118*^ larvae, the methyltransferase activity using FA as substrate was at least 10-fold higher than that using JHA (Fig. 2A). In *jhamt* ^*2*^ larvae, the activity of methyltransferase using JHA as the substrate was similar to that of wild-type glands, whereas the activity of methyltransferase using FA as the substrate decreased by 90% when compared to that in wild-type glands (Fig. 2A).

Using the radiochemical assay followed by thin layer chromatography analysis, we studied the biosynthesis of JHB3, JH III, and MF by the brain-RG complexes dissected from 3h AIW larvae. As previously reported [2, 18, 19], JHB3 was the most abundant product released by wild-type glands, the amount of MF released was about half that of JHB3, whereas JH III was produced at the lowest rate. Remarkably, although JHB3 biosynthesis in *jhamt* ^*2*^ larval glands decreased by 75% when compared to that in wild-type glands, the rates of JH III and MF biosynthesis were not affected (Fig. 2B).

Finally, using a recently developed HPLC-FD protocol [34], we measured whole body titers of JHB3, JH III, and MF in 3h AIW larvae. In *w* ^*1118*^ larvae, MF was the most abundant sesquiterpenoid (~670 fmol/larva), followed by JHB3 (~18 fmol/larva) and JH III (~2.5 fmol/larva) (Fig. 2C–2C”). Although JHB3 showed higher biosynthetic rates, MF showed a higher titer in the larvae, suggesting that MF could be more stable than JHB3 in the body. Whole body titers of JHB3, JH III, and MF in *jhamt* ^*2*^ larvae decreased by approximately 70%, 50%, and 30% (no statistical difference) to their respective control levels (Fig. 2C–2C”). Our data thus suggest that 1) *jhamt* is critical for JHB3 biosynthesis, but not for the biosynthesis of MF and JH III, and 2) the highly abundant MF might play important roles during *Drosophila* metamorphosis.

### Decrease in biosynthesis and titers of the three sesquiterpenoids result in complete lethality

To better understand the relation between the JH-deficient lethal phenotypes and the biosynthesis of the three sesquiterpenoids by the larval CA, we further explored the effect of additional loss-of-function of enzymes in the JH biosynthetic pathway. *Drosophila* CG10527 is an ortholog of a crustacean FA methyltransferase [35], which has been reported as not involved in JH biosynthesis in *Drosophila* [33, 36]. We generated a *jhamt CG10527* double mutant, *jhamt* ^*2*^ *CG10527* ^*187*^ (S3 Fig). Mutation of *CG10527* in a *jhamt* ^*2*^ background did not increase JH-deficient phenotypes (S4 Fig), confirming that CG10527 is not involved in FA or JHA methylation in *Drosophila*.

**Fig 3:**
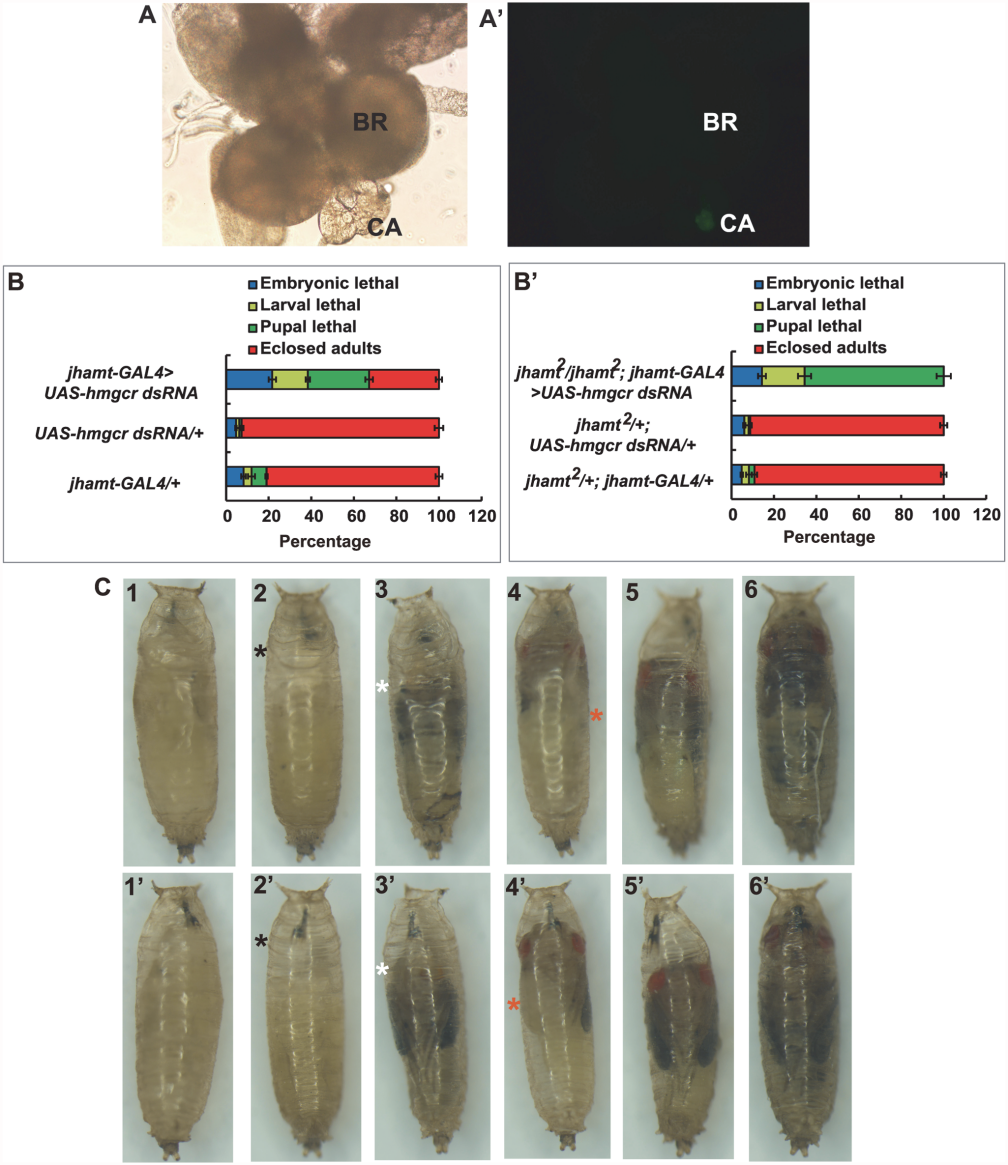
Reduction of *hmgcr* expression in the CA of *jhamt* mutant results in complete lethality. (A and A’) The brain-RG complex in *jhamt-GAL4>UAS-GFP*. BR, brain; CA, corpus allatum. Observed under bright-field (A) or fluorescence (A’) using the same microscope. The CA cells expressing JHAMT were labeled with GFP. (B and B’) (B) Lethality of *jhamt-GAL4*>*UAS-hmgcr dsRNA* during the embryonic, larval, and pupal stages. *jhamt-GAL4/+* and *UAS-hmgcr dsRNA/+* were used as the controls. (B’) Lethality of *jhamt* ^*2*^/*jhamt* ^*2*^; *jhamt-GAL4>UAS-hmgcr dsRNA* during the embryonic, larval, and pupal stages. *jhamt* ^*2*^/+; *jhamt-GAL4/+* and *jhamt* ^*2*^ */+; UAS-hmgcr dsRNA/+* were used as the controls. (C) Images of various pupal lethal phenotypes of *jhamt* ^*2*^ */jhamt* ^*2*^; *jhamt-GAL4>UAS-hmgcr dsRNA*. (1–6) the abdominal sides; (1’-6’) the dorsal sides. The black asterisks point to empty portions of the pupae; the white asterisks, eye defects showing no pigmentation; the red asterisks, wing defects showing a unilateral wing loss.

Different promoters can be used to drive CA-specific expression in *Drosophila*. We have previously shown that *jhamt-GAL4* has a more robust CA-specific expression than *Aug21-GAL4* [21]. Therefore, we generated *jhamt-GAL4>UAS-GFP* flies, which exhibited strong CA-specific expression of GFP (Fig. 3A and 3A’). As expected, similar to *jhamt* ^*2*^ */jhamt* ^*2*^; *Aug21-GAL4*>*UAS-jhamt*, fecundity and ovary growth of *jhamt* ^*2*^ */jhamt* ^*2*^; *jhamt-GAL4*>*UAS-jhamt* were restored to levels similar to those in *w* ^*1118*^ (S2D and S2D’ Fig). We then generated *Aug21-GAL4*>*UAS-hmgcr dsRNA* and *jhamt-GAL4*>*UAS-hmgcr dsRNA* animals, in which *hmgcr* expression is specifically reduced in the CA by RNAi. As detected by quantitative real-time PCR (qPCR), *hmgcr* expression in the brain-RG complexes at 3h AIW decreased by ~35% in *Aug21-GAL4*>*UAS-hmgcr dsRNA* animals and ~50% in *jhamt-GAL4*>*UAS-hmgcr dsRNA* animals (S5A Fig). Lethality of ~55% and ~70% was observed in *Aug21-GAL4*>*UAS-hmgcr dsRNA* (S5B Fig) and *jhamt-GAL4*>*UAS-hmgcr dsRNA* animals (Fig. 3B), respectively. Moreover, the lethality in *jhamt* ^*2*^ */jhamt* ^*2*^; *Aug21-GAL4>UAS-hmgcr dsRNA* was about 93% (S5C Fig), whereas 100% lethality before adult emergence was observed in *jhamt* ^*2*^ */jhamt* ^*2*^; *jhamt-GAL4>UAS-hmgcr dsRNA* (Fig. 3B’). Most *jhamt* ^*2*^ */jhamt* ^*2*^; *jhamt-GAL4>UAS-hmgcr dsRNA* animals died during the pupal stage (60%), exhibiting a variety of developmental defects (Fig. 3C). These data not only confirmed that *jhamt-GAL4* has a more robust CA-specific expression than *Aug21-GAL4*, but also demonstrated that reduction of *hmgcr* expression in the CA in a *jhamt* ^*2*^ background causes stronger lethal phenotypes than the *jhamt* mutant alone.

**Fig 4:**
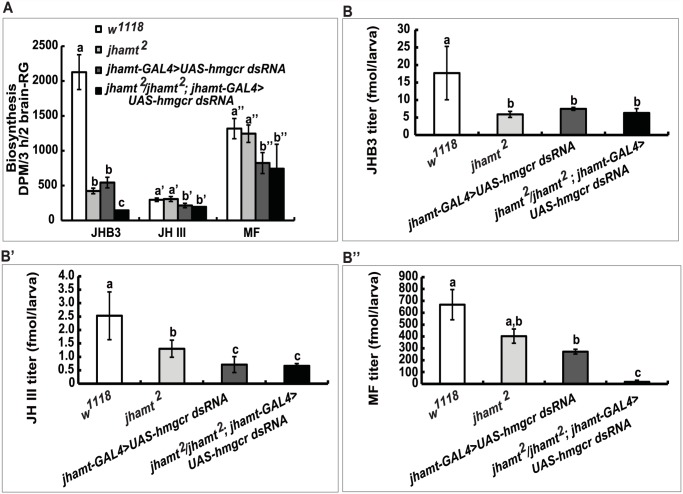
Reduction of *hmgcr* expression in the CA of *jhamt* mutant dramatically decreases biosynthesis and titers of the three sesquiterpenoids. Measurements of biosynthesis of JHB3, JH III, and MF in the brain-RG complexes (A) and whole body titers of JHB3 (B), JH III (B’), and MF (B”) titer in *w* ^*1118*^, *jhamt* ^*2*^, *jhamt-GAL4*>*UAS-hmgcr dsRNA*, and *jhamt* ^*2*^/*jhamt* ^*2*^ *; jhamt-GAL4>UAS-hmgcr dsRNA* at 3h AIW.

Overall, these experiments suggest that reduction of *hmgcr* expression in the CA in a *jhamt* ^*2*^ background decreases biosynthesis and titers of the three sesquiterpenoids to very low levels, resulting in complete lethality. In the following experiments, *jhamt-GAL4*>*UAS-hmgcr dsRNA* (*hmgcrRNAi*) and *jhamt* ^*2*^ */jhamt* ^*2*^; *jhamt-GAL4>UAS-hmgcr dsRNA* (*jhamt* ^*2*^ *hmgcrRNAi*) were used to further confirm the above hypothesis. We measured JH biosynthesis in larval brain-RG complexes isolated from four different lines at 3h AIW: *w* ^*1118*^, *jhamt* ^*2*^, *hmgcrRNAi*, and *jhamt* ^*2*^ *hmgcrRNAi*. In comparison with the *w* ^*1118*^ larvae, JHB3 biosynthesis decreased by 75% in *jhamt* ^*2*^ and *hmgcrRNAi* larvae and by more than 90% in *jhamt* ^*2*^ *hmgcrRNAi* larvae. JH III biosynthesis was not altered in *jhamt* ^*2*^ larvae, but decreased by 30–40% in *hmgcrRNAi* and *jhamt* ^*2*^ *hmgcrRNAi* larvae. MF biosynthesis was not altered in *jhamt* ^*2*^ larvae, but decreased to about 50% in *hmgcrRNAi* and *jhamt* ^*2*^ *hmgcrRNAi* larvae (Fig. 4A).

We also measured titers of the three sesquiterpenoids in the whole larval bodies of the four above mentioned genotypes at 3h AIW. In comparison with the *w* ^*1118*^ larvae, JHB3 titer decreased by 60–70% in *jhamt* ^*2*^, *hmgcrRNAi*, and *jhamt* ^*2*^ *hmgcrRNAi* larvae (Fig. 4B). JH III titer decreased by 50% in *jhamt* ^*2*^ larvae, whereas it decreased by 70–75% in *hmgcrRNAi* and *jhamt* ^*2*^ *hmgcrRNAi* larvae (Fig. 4B’). MF titer decreased by 30% (not statistically significant difference) in *jhamt* ^*2*^ larvae, whereas the decrease was approximately 40% in *hmgcrRNAi* larvae (Fig. 4B”). Interestingly, MF titer decreased by 98% in *jhamt* ^*2*^ *hmgcrRNAi* larvae (Fig. 4B”), implying that most of MF is converted to JHs in *jhamt* ^*2*^ *hmgcrRNAi* larvae. Overall, these experiments suggest that the three sesquiterpenoids synthesized and released by the larval CA are required for *Drosophila* to survive to adulthood; in particular, that the very abundant MF plays essential anti-metamorphic roles during *Drosophila* development (Table 1).

**Table 1:** Comparisons of JH biosynthesis, JH titer, and lethality among three genotypes.

	Biosynthesis			Titer			Lethality
	JHB3	MF	JH III	JHB3	MF	JH III	
*jhamt* ^*2*^	↓	No change	No change	↓	No change	↓	No
*jhamt-GAL4>UAS-hmgcr dsRNA*	↓	↓	↓	↓	↓	↓↓	Partial
*jhamt* ^*2*^/*jhamt* ^*2*^ *; jhamt-GAL4> UAS-hmgcr dsRNA*	↓↓	↓	↓	↓	↓↓	↓↓	Complete

### MF acts through Met/Gce to induce *Kr-h1* expression and prevents lethality of JH-deficient flies but not *Met gce* double mutant

**Fig 5:**
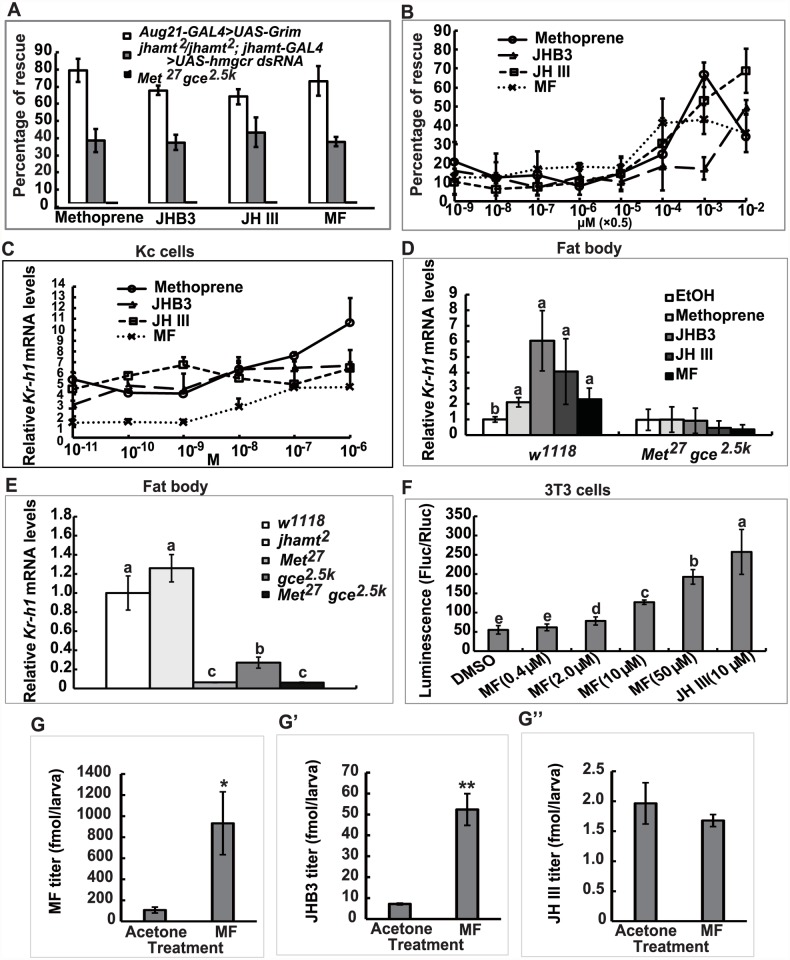
MF plays a dual role: as a JHB3 precursor and as a hormone. (A) Percentage of rescuing *Aug21-GAL4>UAS-Grim*, *jhamt* ^*2*^/*jhamt* ^*2*^ *; jhamt-GAL4>UAS-hmgcr dsRNA*, and *Met* ^*27*^ *gce* ^*2*.*5k*^ to adults by topical application of methoprene, JHB3, JH III and MF (0.5×10^-2^ μmol per larva) at 96h AEL. (B) Percentage of rescuing *jhamt* ^*2*^/*jhamt* ^*2*^ *; jhamt-GAL4>UAS-hmgcr dsRNA* to adults by topical application of a dose gradient of methoprene, JHB3, JH III, and MF (0.5×10^-9~-2^ μmol per larva) at 96h AEL. (C) qPCR measurements of fold-changes of relative *Kr-h1* mRNA levels in Kc cells treated with methoprene, JHB3, JH III, and MF (1×10^-10~-6^ M) for 30 min. (D) qPCR measurements of relative *Kr-h1* mRNA levels in fat body tissues isolated from *w* ^*1118*^ and *Met* ^*27*^ *gce* ^*2*.*5k*^ at 96h AEL after treatments with methoprene, JHB3, JH III, and MF (1×10^-6^ M) for 30 min. (E) qPCR measurements of the relative *Kr-h1* mRNA levels in the fat body tissues isolated from *w* ^*1118*^, *jhamt* ^*2*^, *Met* ^*27*^, *gce* ^*2*.*5k*^, and *Met* ^*27*^ *gce* ^*2*.*5k*^ at 3h AIW. (F) MF promotes interaction of Met and SRC in mouse embryonic fibroblast 3T3 cells. 3T3 cells were transiently transfected with GAL4:TcMet and TcSRC. And the transfected cells were cultured in the medium containing different concentrations of MF and JH III (DMSO as control). After 24 hours exposure to the ligands, cells were assayed for luciferase reporter activity. The luciferase activity was normalized based on the total protein concentration determined for cells in each well. (G-G”) Measurements whole body titers of JHB3 (G), JH III (G’), and MF (G”) in *jhamt* ^*2*^/*jhamt* ^*2*^ *; jhamt-GAL4>UAS-hmgcr dsRNA* at 3hAIW after topical application of MF (0.5×10^-2^ μmol per larva; dissolved in acetone) at 96h AEL (about 24 hours after treatments).

To further understand the anti-metamorphic roles of each of the three sesquiterpenoids synthesized by the larval CA, we performed a series of experiments by treating JH-deficient animals with methoprene or sesquiterpenoids to evaluate their ability to prevent lethality, as well as their efficiency in inducing expression of the JH-responsive gene *Kr-h1*. Topical application of high doses of methoprene, JHB3, JH III, and MF (0.5×10^-2^ μmol per larva) to third instar larvae when JH titers are low (at 96h AEL: 96 hours after egg laying) [10] was able to decrease mortality significantly (40–75%) in the two JH-deficient animals (*Aug21>Grim* and *jhamt* ^*2*^ *hmgcrRNAi*). By contrast, neither methoprene nor sesquiterpenoids (0.5×10^-2^ μmol per larva) prevented the lethality of *Met* ^*27*^ *gce* ^*2*.*5k*^ (Fig. 5A). Additional experiments were performed on *jhamt* ^*2*^ *hmgcrRNAi* to evaluate the dose-responses for methoprene and the three sesquiterpenoids in preventing lethality. These compounds showed significant effects at 0.5×10^-4^ μmol/larva, with MF being the most effective, followed by JH III, methoprene, and JHB3. At higher doses (0.5×10^-3^ and 0.5×10^-2^ μmol/larva), only the effects of JHB3 and JH III continued to increase (Fig. 5B).

qPCR was utilized to examine whether MF acts through Met/Gce to induce *Kr-h1* expression [20, 30, 31]. Methoprene and the three sesquiterpenoids induced *Kr-h1* expression in both *Drosophila* Kc cells (1×10^-10~-6^ M) (Fig. 5C) and cultured fat body tissues isolated from *w* ^*1118*^ larvae at 96h AEL (1×10^-7^ M) (Fig. 5D, left panel); although induction with MF was weaker than JHB3 and JH III. We also determined *Kr-h1* mRNA levels in *jhamt* ^*2*^ larvae, wherein JHB3 biosynthesis but not MF biosynthesis is reduced (Fig. 2A). *Kr-h1* expression was normal in 3h AIW *jhamt* ^*2*^ larvae, indicating that the other two sesquiterpenoids (in particular the very abundant MF) were sufficient to induce *Kr-h1* expression to control levels. In contrast, as previously reported [21], in *Met* ^*27*^ *gce* ^*2*.*5k*^ larvae, *Kr-h1* mRNA levels were reduced by about 95% when compared to its levels in *w* ^*1118*^ larvae (Fig. 5E). As expected, methoprene and the three sesquiterpenoids failed to induce *Kr-h1* expression in cultured fat body tissues isolated from *Met* ^*27*^ *gce* ^*2*.*5k*^ larvae at 96h AEL (Fig. 5D, right panel). These data from *in vitro* and *in vivo* experiments revealed that, in addition to JHB3 and JH III, MF also has an anti-metamorphic or “JH-like” role in *Drosophila* larvae, acting through Met/Gce to induce *Kr-h1* expression.

We then extended our study to *Tribolium*, in which JH III directly induces heterodimerization of the JH receptor (TcMet) and its partner (TcSRC) in mouse embryonic fibroblast 3T3 cells [37]. Here we found that MF also induced heterodimerization of TctMet and TcSRC in 3T3 cells in a dose-dependent manner, although its induction ability was weaker than JH III (Fig. 5F). This experiment provides strong evidence that MF acts as a hormone itself through a direct interaction with the JH receptor Met in *Tribolium*, supporting the above findings in *Drosophila*.

### MF plays a dual role: As a JHB3 precursor and as a hormone

**Fig 6:**
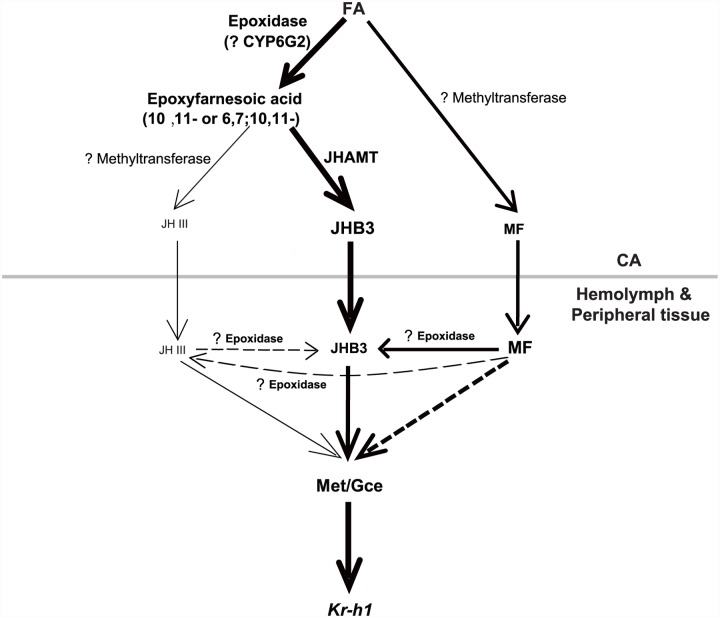
A possible model showing the last two steps of biosynthesis and the molecular actions of the three sesquiterpenoids in *Drosophila*. In the CA, FA is the common precursors for JHB3, JH III, and MF biosynthesis. In the hemolymph and peripheral tissues, MF either directly acts through Met/Gce or is converted to JHB3. JHAMT only accounts for JHB3 biosynthesis; and other methyltransferases and P450 epoxidase with question marks have not been identified. Please see Discussion for details on the model. Text and arrow sizes convey magnitude of treatment and response. The gray line separates CA from hemolymph and peripheral tissues.

Finally, we examined whether once released by the CA, MF could be converted to JHB3 or JH III in the fly hemolymph or peripheral tissues. The *jhamt* ^*2*^ *hmgcrRNAi* larvae were topically treated with acetone or MF (0.5×10^-2^ μmol per larva) at 108h AEL, and the three sesquiterpenoid titers were measured at 3h AIW (about 24 hours after treatment). While JH III titer did not change, MF and JHB3 titers in the MF-treated animals increased approximately 9- and 7-fold respectively when compared to control animals treated with acetone (Fig. 5G–5G”). The topical application experiments showed that a portion of the exogenous MF was converted to JHB3 in the hemolymph or peripheral tissues, consistent with the results obtained from *jhamt* ^*2*^ (Fig. 2C–2C”) and *jhamt* ^*2*^ *hmgcrRNAi* larvae (Fig. 4B–4B”). We conclude that MF is required for completion of *Drosophila* metamorphosis, playing a dual role: as a JHB3 precursor and as a hormone (Fig. 6).

## Discussion

### Requirement of the three sesquiterpenoids for completion of *Drosophila* metamorphosis

This study (Table 1; Figs 1–4) confirmed and expanded previous studies, showing that genetic ablation of the CA caused JH deficiency and pupal lethality in *Drosophila* [20, 22]. Knockdown and/or knockout of enzymes in the early and late steps of the JH biosynthetic pathway generated different phenotypes depending on the background of the animals: 1) null mutation of *jhamt* resulted in significant decrease in JHB3 biosynthesis, as well as JHB3 and JH III titers, without compromising development and survival, 2) RNAi-mediated reduction of *hmgcr* expression in the CA decreased biosynthesis and titers of the three sesquiterpenoids produced by the larval CA, resulting in partial lethality, and 3) RNAi-mediated reduction of *hmgcr* expression in the CA of the *jhamt* mutant further decreased JHB3 biosynthesis and MF titer, leading to complete lethality. These results lead us to conclude that only dramatic decreases in biosynthesis of the three sesquiterpenoids resulted in very low circulating titers and caused complete lethality in the two JH-deficient animals (*Aug21>grim* and *jhamt* ^*2*^ *hmgcrRNAi*). Moreover, the requirement of the three sesquiterpenoids for *Drosophila* metamorphosis was further strengthened by the rescue experiments in the two JH-deficient animals (Fig. 5A and 5B), showing that JHB3, JH III, and MF are able to functionally replace one another.

### MF plays a dual role: As a JHB3 precursor and as a hormone during *Drosophila* metamorphosis

Although accepted as the anti-metamorphic hormone in Crustacea, the potential role of MF as a true JH in Insecta has been an issue of a long-standing debate [1, 4, 24, 37]. Our experiments provide additional evidence that supports the anti-metamorphic or “JH-like” role of MF in *Drosophil*a, including: 1) the fact that MF is released by the CA and is the most abundant sesquiterpenoid present in extracts of larval body, 2) the ability to phenocopy anti-metamorphic roles following topical application to JH-deficient animals (“rescue” experiments), 3) the capability to act through the JH receptors (Met and Gce) and induce a dose-dependent expression of *Kr-h1*, a JH primary-response anti-metamorphic gene, and 4) the conversion to JHB3 in the hemolymph or peripheral tissues.

The presence of high circulating MF levels has been previously described in *Drosophila* larvae [9, 10], as well as the production of MF by the larval brain-RG complexes [3]. MF might also play an anti-metamorphic role during early larval development in *Bombyx*; high levels of MF might exist in *Bombyx dimolting*, a P450 epoxidase mutant, that contains no detectable JH I, JH II, and JH III in the hemolymph [16].

The ability of MF to phenocopy anti-metamorphic roles has been previously established in the white puparia JH bioassay [6, 7]. The importance of MF during *Drosophila* metamorphosis was validated by the RNAi-mediated reduction of *hmgcr* expression in the CA of the *jhamt* mutant, in which only MF was further decreased leading to complete lethality (Table 1); as well as by the observation that JHB3, JH III and MF efficiently precluded lethality in two JH-deficient lines.

It has been suggested that MF could play anti-metamorphic roles acting through ultraspiracle (USP, an ortholog of the retinoid X receptor and a molecular partner of the 20E receptor, EcR) [9]. On the other hand, MF efficiently competes with JH III for binding to Met and Gce in *Drosophila* [28], MF directly induces heterodimerization of Met and SRC of Crustacea in mammalian cells [38], and MF induces *Kr-h1* promoter activity in mammalian cells in the presence of *Bombyx* Met and SRC [39]. We validated and expanded those results, showing that MF induces a dose-dependent *Kr-h1* expression in *Drosophila* cell lines and fat body tissues isolated from JH-deficient animals (Fig. 5C–5E). Moreover, MF induces heterodimerization of Met and SRC of *Tribolium* in mammalian 3T3 cells in a dose-dependent manner (Fig. 5F). Data included in this paper show that MF acts through Met/Gce (Fig. 5C–5F), but not USP (S6 Fig), at least in the induction of *Kr-h1* expression and Met-SRC heterodimerization.

Finally we showed that MF can be converted in the hemolymph or peripheral tissues to other active JHs in *Drosophila*. In *jhamt* ^*2*^ larvae, JHB3 biosynthesis is dramatically reduced and MF and JH III biosynthesis are unaffected (Fig. 2B), whereas whole body titers of JHB3, JH III, and MF decreased by approximately 70%, 50%, and 30% (no statistical difference) relative to their respective control levels (Fig. 2C–2C”). The decrease in whole body levels of MF could be the consequence of a portion of the MF pool undergoing conversion to JHB3 in *jhamt* ^*2*^ larvae. In comparison with *hmgcrRNAi* larvae, JHB3 biosynthesis is further reduced in *jhamt* ^*2*^ *hmgcrRNAi* larvae, whereas the biosynthesis of MF and JH III is unaffected (Fig. 4A). Similarly, although MF titer decreased to almost zero in *jhamt* ^*2*^ *hmgcrRNAi* larvae, JHB3 and JH III titers remained at the same levels (Fig. 4B–4B”), suggesting again that most of MF is converted to JHB3 in *jhamt* ^*2*^ *hmgcrRNAi* larvae. The possibility that MF can be converted to other JHs was further confirmed by topical application of MF to *jhamt* ^*2*^ *hmgcrRNAi* larvae (Fig. 5G–5G”).

We conclude that MF plays a dual role in regulating *Drosophila* metamorphosis: through its conversion to JHB3, as well as through its role as a *bona fide* juvenoid (Fig. 6).

Was MF the ancestral ‘JH’ of Arthropods? Ongoing studies of the metabolic pathways for JH biosynthesis and degradation in other Arthropods, including Myriapods and Chelicerates, indicate that these groups all possess the requisite enzymes to produce at least MF. In particular, these groups all appear to possess a JHAMT ortholog, indicating that MF may have been synthesized and functional in these groups. These groups also possess enzymes known to be involved in the degradation of the sesquiterpenoids, as well as binding proteins [40, 41]. At present, it is unknown if these groups possess a functional member of the CYP family of cytochrome P450 enzymes that would be responsible for the epoxidation of MF. The apparent absence of this enzyme in crustaceans and possibly in *Drosophila* argues for the importance of MF in the regulation of metamorphosis. These studies suggest that the ‘JH’ signaling pathway has deep evolutionary roots [40, 41] and our present results on *Drosophila* support such a view. These authors also suggest that the pathway “might have evolved together with the emergence of the exoskeleton”. This suggestion highlights the importance of MF, particularly in metamorphosis. During evolution in arthropods, MF maintains its anti-metamorphic role from crustaceans to insects and probably across the phylum. Subsequently, different JHs emerged in different orders of insects. Diversification of the JH(s) might contribute to variation and novelty during arthropod evolution. The co-existence of three JHs and two JH receptors in a single organism makes *Drosophila* a complicated but fascinating system for studying the JH signal transduction pathway, from both molecular and evolutionary perspectives.

### The last two steps of JH biosynthesis in *Drosophila*

Compared with other insects producing only JH III, the last two steps of the JH biosynthetic pathway in *Drosophila* are much more ambiguous. We propose a JH biosynthetic pathway in which FA is the common precursor for JHB3, JH III, and MF in *Drosophila* (Fig. 6). Our previous studies [19] and the data included in this paper (Fig. 2) show that overexpression and mutation of *jhamt* increased and decreased JHB3 biosynthesis, respectively, but did not affect the production of JH III and MF, suggesting that JHAMT is responsible only for JHB3 biosynthesis in the CA. Moreover, mutation of *jhamt* significantly decreased the activity of methyltransferase using FA but not JHA as substrate, implying the existence of one or more additional methyltransferases converting FA into MF and JHA into JH III in the CA of *Drosophila* larvae.

It has been suggested that the lack of a clear ortholog of a P450 epoxidase in *Drosophila* might be explained on the basis of the different chemistry of the fly JHs [15]. The CYP15 of higher flies could have evolved to allow the epoxidation at both the 6, 7 and 10, 11 double bonds, and this evolution resulted in such significant changes so that the sequence is no longer recognizable as a CYP15. A global analysis of CYP enzymes in *Drosophila* revealed specific expression of CYP6G2 in the CA [42], but whether it functions as a P450 epoxidase is currently unknown. One possibility is that CYP6G2 preferably epoxidizes FA to 6, 7; 10, 11-epoxyfarnesoic acid (JHB3 acid) rather than 6, 7-epoxyfarnesoic acid (JHA), resulting in a much higher JHB3 biosynthesis ratio compared to the JH III biosynthesis ratio. Moreover, we found that a portion of MF was converted to JHB3 in the hemolymph or peripheral tissues (Fig. 2, 4, 6), presumably by an uncharacterized P450 epoxidase. The identification of the methyltransferases and P450 epoxidases that are involved in the last two steps of JH biosynthesis in *Drosophila* remains as a future challenge.

## Materials and Methods

### Flies and genetics

To generate the *jhamt* mutant, we used the homologous recombination—mediated ends-out gene targeting technique [32]. Two genomic DNA fragments flanking the *jhamt* (*CG17330*) coding region were amplified by PCR. The upstream flanking region (4245-bp length: -4212 bp to +33 bp from the translational start site of *jhamt*) was cloned into the *pw25* plasmid using the *Not* I (*jhamt-5’end-Not* I) and *Acc65* I (*jhamt-5’end-Acc65* I) restriction sites introduced by PCR primers. Subsequently, the downstream flanking region (3977-bp length: +1050 bp to +5027 bp from the start site of the *jhamt* gene) was cloned into the above generated vector using the *Asc* I (*jhamt-3’end-Asc* I) and *BsiW* I (*jhamt-3’end-BsiW* I) restriction sites. The resulting construct of *pw25-jhamt* (Fig. 1A) was used to generate transgenic flies using P-element-mediated germline transformation. Then, the *pw25-jhamt* transgenic flies were crossed with *yw*; *p{70FLP}23 p{70I-Sce*I*}4A/TM6* to generate the *jhamt* knock-out strains (*jhamt* ^*1*^, *jhamt* ^*2*^, and *jhamt* ^*3*^) (Fig. 1B and 1C). Primers used here and elsewhere are listed in S1 Table.

The putative promoter sequence (2540-bp length: -2544 bp to-4 bp, from the translational start site of *jhamt*) of *jhamt* was amplified as a *Sac* II*-BamH* I fragment, and cloned into the *pChsGAL4* plasmid to generate the *jhamt-GAL4* construct. The *jhamt-GAL4* transgenic flies were then produced.

*w* ^*1118*^, *Aug21-GAL4*, *Act-GAL4*, *UAS-GFP*, *UAS-grim*, *CG10527* ^*187*^, *Met* ^*27*^, *gce* ^*2*.*5k*^, and *Met* ^*27*^ *gce* ^*2*.*5k*^ were reported previously [14, 20, 21, 31, 33]. Multiple *UAS-hmgcr dsRNA* lines (stock number 11635 is reported) were obtained from the Vienna *Drosophila* RNAi Center. RNAi lines were also obtained from the Bloomington *Drosophila* Stock Center, and similar results were obtained. Other flies used in this paper were generated by recombination. All fly strains in this paper were grown at 25°C on standard cornmeal/molasses/agar medium.

### PCR and western blot analysis

For genomic DNA PCR, genomic DNA was extracted from flies using phenol-chloroform-isoamyl alcohol. To confirm the *jhamt* mutants and the *jhamt* ^*2*^ *CG10527* ^*187*^ double mutants, genomic DNA PCR was performed with 4 primer pairs, including *jhamt-1* and *jhamt-2* (689-bp length), *jhamt-3* and *jhamt-4* (812-bp length), *jhamt-1* and *jhamt-5* (671-bp length), and *jhamt-6* and *jhamt-4* (1259-bp length) (Fig. 1A and 1B). To identify and confirm the *CG10527* ^*187*^ mutation in the *jhamt* ^*2*^ *CG10527* ^*187*^ double mutant, genomic DNA PCR were performed with primer pairs *CG10527-F* and *CG10527*-*R* (1968-bp for wild type and ~600-bp for the *CG10527* ^*187*^ mutant) (S3 Fig). For reverse transcription PCR, a primer pair *jhamt*-7 and *jhamt-8* (405-bp) were used to detect *jhamt* mRNA expression from the brain-RG complexes isolated from larvae at 3hAIW (Fig. 1C). qPCR was performed as previously described [14, 20, 21, 31, 33].

DmCG10527 rat polyclonal antibody [33] was used to conduct the Western blot analysis of the brain-RG complexes isolated from larvae at 3hAIW. The tubulin mouse monoclonal antibody (#AT819, Beyotime, China) was used as an internal control.

### Immunohistochemistry

For detecting JHAMT in the CA by immunohistochemistry, the brain-RG complexes were dissected from larvae at the EW stage. The *Drosophila* JHAMT rabbit polyclonal antibody (1:100) [13] and the FITC-conjugated Affinipure Goat Anti-Rabbit IgG secondary antibody (Jackson ImmunoResearch Inc.) were used, and the fluorescence signals were captured with an Olympus IX71 invert fluorescence microscope (Japan) [14, 20, 31].

### JH treatments and cell culture

Methoprene (Service Chemical Inc., Germany), JH III (Sigma-Aldrich), and MF (Echelon) were purchased. JHB3 was synthesized from MF using m-chloroperbenzoic acid in dichloromethane (Sigma-Aldrich) [19]. For rescue of fertility of *jhamt* ^*2*^, newly eclosed females were placed in vials with standard medium; after 24 hours, virgin females were topically treated with acetone-dissolved methoprene (0.5 μl × 10^-3^ M per female) [21, 23]. For rescue of pupal lethality of *Aug21>grim* and *jhamt* ^*2*^ *hmgcrRNAi*, methoprene, JHB3, JH III, and MF (0.5 μl × 10^-9~-2^ M per larva) were dissolved in acetone and topically applied to the larvae at 96h AIW [14, 20, 21, 31, 33]. For inducing *Kr-h1* expression in *w* ^*1118*^ and *Met* ^*27*^ *gce* ^*2*.*5k*^, fat body tissues were isolated at 96h AIW and treated with methoprene, JHB3, JH III, and MF (1×10^-6^ M; DMSO as a control) for 30 min. For testing the conversion of MF to other JHs, the *jhamt* ^*2*^ *hmgcrRNAi* larvae were topically treated with acetone or MF (0.5×10^-2^ μmol per larva) at 108h AEL, and the three sesquiterpenoids titers were measured at 3hAIW (about 24 hours after treatment).

For inducing *Kr-h1* expression in *Drosophila* Kc cells cultured in Schneider’s medium, the cells were treated with methoprene, JHB3, JH III, and MF (1×10^-11~-6^ M; DMSO as a control) for 30 min [31]. Using the T7 RiboMAX Express RNAi System (Promega), dsRNAs of *USP* and *EGFP* (as a control) were synthesized. Reduction of gene expression by RNAi in Kc cells was performed by transfecting dsRNAs using Effectene at a final concentration of 1 μg/ml dsRNA. The transfected cells were cultured for 48 h and treated with MF (1×10^-6^ M; DMSO as a control) for 30 min [31].

### Luciferase assay in 3T3 cells

3T3 cells were grown at 37°C with 5% CO2 in a DMEM (life technology) containing 10% fetal bovine serum. For transfection experiments, 50,000 cells/well were seeded in a 48-well plate. On the following day, the cells were transiently transfected with 67 ng each of receptor/partner and 200 ng each of pFRLUC reporter construct, using a “Polyfect” transfection reagent (Qiagen). After 4 hours, different final concentration of MF (0.4, 2, 10 and 50 μM) were added to the wells along with DMEM medium with 20% FBS as well. DMSO and 10 μM JH III were used as a negative and positive control, respectively. After 24 hours exposure to the ligands, cells were washed with PBS, 60 μl of reporter lysis buffer was added to each well and luciferase reporter activity was measured using the luciferase reporter assay system from Promega (Madison, WI). To standardize the luciferase activity, protein concentration in cells from well was determined using the Bradford reagent. Details on the constructs GAL4:TcMet in the pBIND vector and TcSRC in the pACT vector, as well as JH III treatment experiments were published previously [37].

### Measurements of methyltransferase activity, JH biosynthesis, and JH titer

S-Adenosyl-L-methionine (SAM) was purchased from Sigma-Aldrich and S-Adenosyl-L-[methyl-^3^H] methionine (370GBq mmol, 10 Ci/mmol) from Perkin-Elmer Life Sciences (Waltham). Methyltransferase activity in the brain-RG complexes isolated from larvae at 3hAIW was measured with JHA and FA as substrates, as described previously [14, 20, 23, 33]. L-[Metyl-^3^H] methionine (2.92–3.70 TBq/mmol) was purchased from Perkin-Elmer Life Sciences and TLC plates (20×20 cm^2^ plastic plate coated with silica gel F254) from Merck KgaA (Germany). JH biosynthesis in the brain-RG complexes was detected using the radiochemical assay followed by thin layer chromatography analysis as reported previously [18, 19, 37]. JH titers from the whole bodies of each genotype were determined using the recently developed HPLC-FD protocol [34].

### Statistics

Experimental data were analyzed with the Student’s *t*-test and ANOVA. *t*-test: *, *p*<0.05; **, *p*<0.01. ANOVA: the bars labeled with different lowercase letters are significantly different (*p*<0.05). Throughout the paper, values are represented as the mean ± standard deviation of at least five independent experiments.

## Supporting Information

S1 Fig The scheme of JH III biosynthetic pathway in insects. (TIF)

S2 Fig Phenotypic changes of the *jhamt* mutant. (A and B) Measurements of the periods from egg laying to wandering (A) and the body weights at the white prepupal stage (B) of *w* ^*1118*^ and *jhamt* ^*2*^. (C and C’) Topical applications of acetone (control) and methoprene (0.5×10^-3^ μmol per female) on newly eclosed females of *w* ^*1118*^ and *jhamt* ^*2*^, and measurements of the average number of eggs laid by each pair of flies per 24 hours (C) and the ovary size of 6-day-old virgins (C’). (D and D’) Comparisons of the average number of eggs laid by each pair of flies per 24 hours (D) and the ovary size of 6-day-old virgins (D’) among *w* ^*1118*^, *jhamt* ^*2*^, *jhamt* ^*2*^ */jhamt* ^*2*^; *Aug21-GAL4*>*UAS-jhamt*, and *jhamt* ^*2*^ */jhamt* ^*2*^; *jhamt-GAL4*>*UAS-jhamt*.
(TIF)

S3 Fig Generation of *jhamt^2^ CG10527^187^*. (A) Genomic structures of *CG10527*. *CG10527* ^*187*^ has an intragenic deletion of *CG10527* compared to *w* ^*1118*^ [33]. The black boxes indicate the coding region, whereas the white boxes denote the non-coding exons. The black bars marked with F and R represent the primer pair *CG10527-F/CG10527-R*. The brown line indicates the PCR products (1968 bp length and ~600 bp length) obtained with the above primer pair using the genomic DNA extracted from *w* ^*1118*^ and *CG10527* ^*187*^ as templates, respectively. The blank region denotes the deletion region of *CG10527* in *CG10527* ^*187*^. (B and B’) Three lines of the *jhamt* ^*2*^ *CG10527* ^*187*^ double mutants were confirmed by genomic DNA PCR. (B) The 1968-bp and ~600 bp PCR products were obtained with primer pair *CG10527-F/CG10527-R* (the black bars) from *w* ^*1118*^ and *CG10527* ^*187*^, respectively. (B’) The *white* PCR products of expected sizes with the primer pairs *jhamt-1/jhamt-2* (the red bars) and *jhamt-3/jhamt-4* (the green bars) (as shown in Fig. 1A) as well as the 1968 bp and ~600 bp PCR products with primer pair *CG10527-F/CG10527-R* (the black bars) were obtained in the 3 heterozygous *jhamt* ^*2*^ *CG10527* ^*187*^ lines (lane 1, 2 and 3). In the following experiments, the number 1 homozygous *jhamt* ^*2*^ *CG10527* ^*187*^ double mutant was used. (C) As detected by Western blot analysis, CG10527 was expressed in the brain-RG complexes of *w* ^*1118*^ and *jhamt* ^*2*^ but not those of *CG10527* ^*187*^ and *jhamt* ^*2*^ *CG10527* ^*187*^. Tubulin was used as the internal control. (D) Immunohistochemistry revealed no expression of JHAMT in the CA of *jhamt* ^*2*^ and *jhamt* ^*2*^ *CG10527* ^*187*^, while JHAMT was expressed in the CA of *w* ^*1118*^ and *CG10527* ^*187*^. Arrows indicate the CA.
(TIF)

S4 Fig Mutation of *CG10527* does not enhance JH-associated effects of the *jhamt mutant*. (A and A’) Measurements of methyltransferase activity of the brain-RG complexes in *w* ^*1118*^, *jhamt* ^*2*^, *CG10527* ^*187*^, and *jhamt* ^*2*^ *CG10527* ^*187*^ at 3h AIW using FA (A) or JHA (A’) as substrates. (B) Measurements of JH biosynthesis in the brain-RG complexes in *w* ^*1118*^, *jhamt* ^*2*^, *CG10527* ^*187*^, and *jhamt* ^*2*^ *CG10527* ^*187*^ at 3h AIW using the RCA-TLC method. (C-C”) Quantitative measurements of whole body titers of JHB3 (C), JH III (C’), and MF (C”) in *w* ^*1118*^, *jhamt* ^*2*^, *CG10527* ^*187*^, and *jhamt* ^*2*^ *CG10527* ^*187*^ at 3h AIW according to the HPLC-FD protocol. (D) qPCR measurements of the relative mRNA levels of *Kr-h1* in the fat body tissues isolated from *w* ^*1118*^, *jhamt* ^*2*^, *CG10527* ^*187*^, and *jhamt* ^*2*^ *CG10527* ^*187*^ at 3h AIW. (E and E’) Comparisons of the average number of eggs laid by each pair of flies per 24 hours (E) and the ovary size of 6-day-old virgins (E’) among *w* ^*1118*^, *jhamt* ^*2*^, *CG10527* ^*187*^, and *jhamt* ^*2*^ *CG10527* ^*187*^.
(TIF)

S5 Fig Lethality of *Aug21-GAL4*>*UAS-hmgcr dsRNA* and *jhamt* ^2^/*jhamt* ^2^; *Aug21-GAL4>UAS-hmgcr dsRNA*. (A) RNAi efficiency of *Aug-GAL4*>*UAS-hmgcr dsRNA* and *jhamt-GAL4*>*UAS-hmgcr dsRNA* at 3h AIW. (B) Lethality of *Aug21-GAL4*>*UAS-hmgcr dsRNA* during the embryonic, larval, and pupal stages. *Aug21-GAL4/+* and *UAS-hmgcr dsRNA/+* were used as the controls. (C) Lethality of *jhamt* ^*2*^/*jhamt* ^*2*^; *Aug21-GAL4>UAS-hmgcr dsRNA* during the embryonic, larval, and pupal stages. *jhamt* ^*2*^/+; *Aug21-GAL4/+* and *jhamt* ^*2*^ */+; UAS-hmgcr dsRNA/+* were used as the controls.
(TIF)

S6 Fig Reduction of *USP* expression does not affect JH-induced *Kr-h1* expression. qPCR measurements of fold-changes of relative *USP* (A) and *Kr-h1* (B) mRNA levels in Kc cells in which *USP* expression was reduced by RNAi (GFP RNAi and DMSO as a control) for 48 h, followed with treatments with MF (1×10^-10~-6^ M) for 30 min.
(TIF)

S1 Table Primers used in this paper. (PDF)
