# Correction: Methyl Farnesoate Plays a Dual Role in Regulating *Drosophila* Metamorphosis

**DOI:** 10.1371/journal.pgen.1006559

**Published:** 2017-01-20

**Authors:** Di Wen, Crisalejandra Rivera-Perez, Mohamed Abdou, Qiangqiang Jia, Qianyu He, Xi Liu, Ola Zyaan, Jingjing Xu, William G. Bendena, Stephen S. Tobe, Fernando G. Noriega, Subba R. Palli, Jian Wang, Sheng Li

The affiliation for the third and seventh author is incorrect. Mohamed Abdou and Ola Zyaan are not affiliated with the Department of Entomology, University of Maryland, College Park, Maryland, United States of America.

The correct affiliation is the Department of Entomology, Faculty of Science, Ain Shams University, Cairo, Egypt.
