# Supplementary figures and images for: Methyl Farnesoate Plays a Dual Role in Regulating Drosophila Metamorphosis
Source: PLoS Genet. 2015 Mar 16;11(3):e1005038. doi: 10.1371/journal.pgen.1005038 (PMC4361637; doi:10.1371/journal.pgen.1005038)

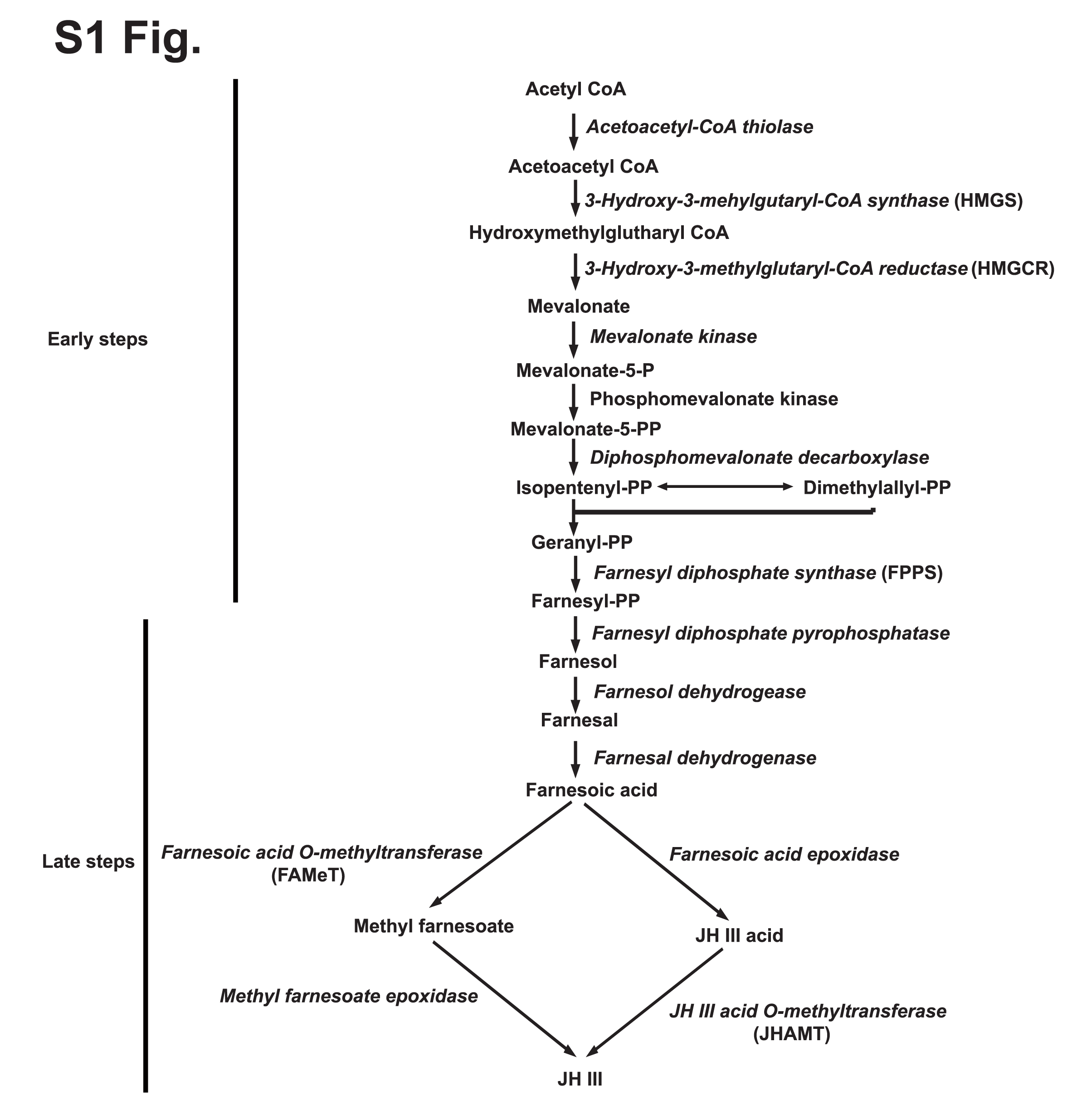

Supplement: S1 Fig — (TIF) [file pgen.1005038.s001.tif]

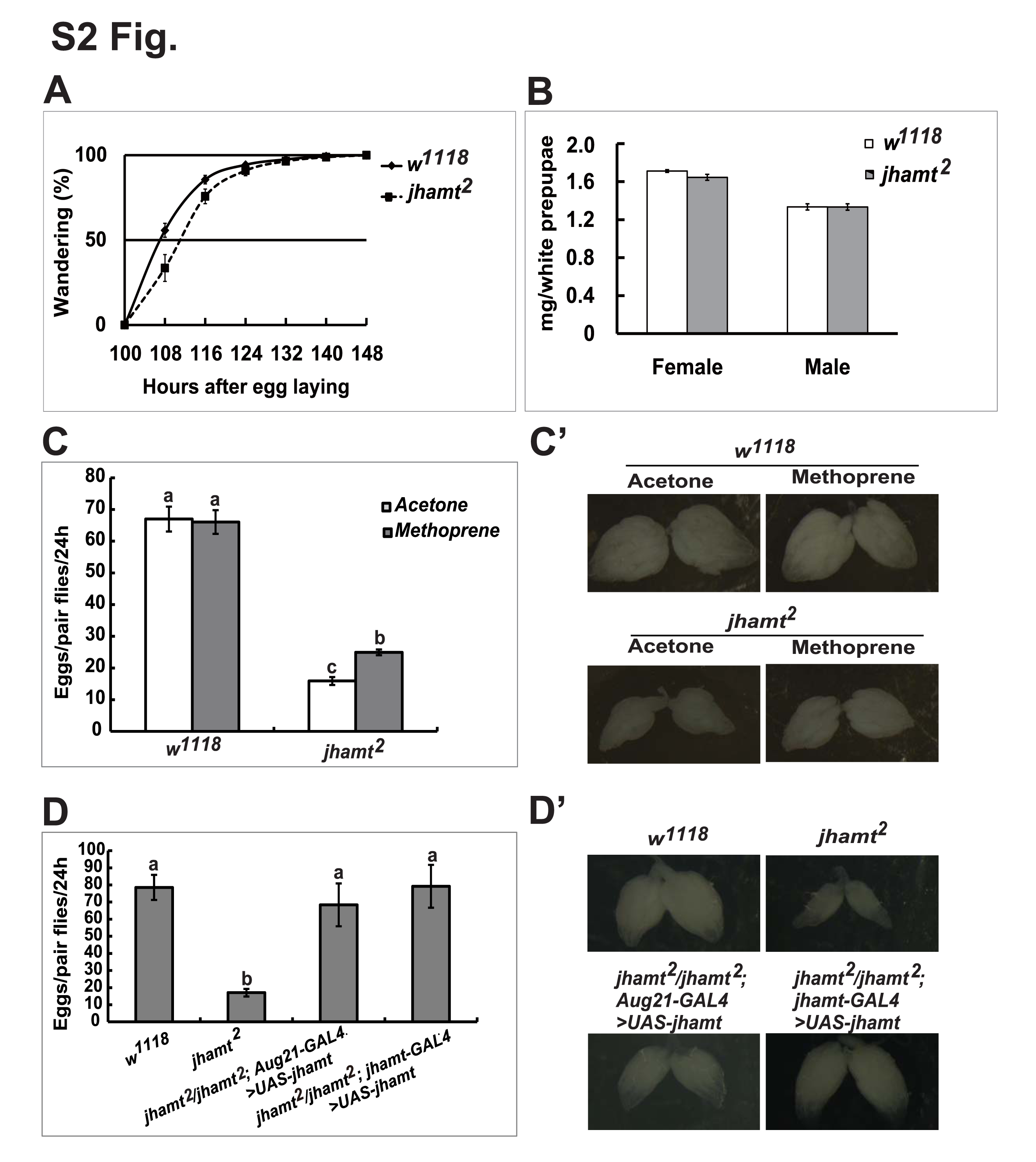

Supplement: S2 Fig — (A and B) Measurements of the periods from egg laying to wandering (A) and the body weights at the white prepupal stage (B) of w 1118 and jhamt 2. (C and C’) Topical applications of acetone (control) and methoprene (0.5×10^-3^ μmol per female) on newly eclosed females of w 1118 and jhamt 2, and measurements of the average number of eggs laid by each pair of flies per 24 hours (C) and the ovary size of 6-day-old virgins (C’). (D and D’) Comparisons of the average number of eggs laid by each pair of flies per 24 hours (D) and the ovary size of 6-day-old virgins (D’) among w 1118, jhamt 2, jhamt 2 /jhamt 2; Aug21-GAL4>UAS-jhamt, and jhamt 2 /jhamt 2; jhamt-GAL4>UAS-jhamt. (TIF) [file pgen.1005038.s002.tif]

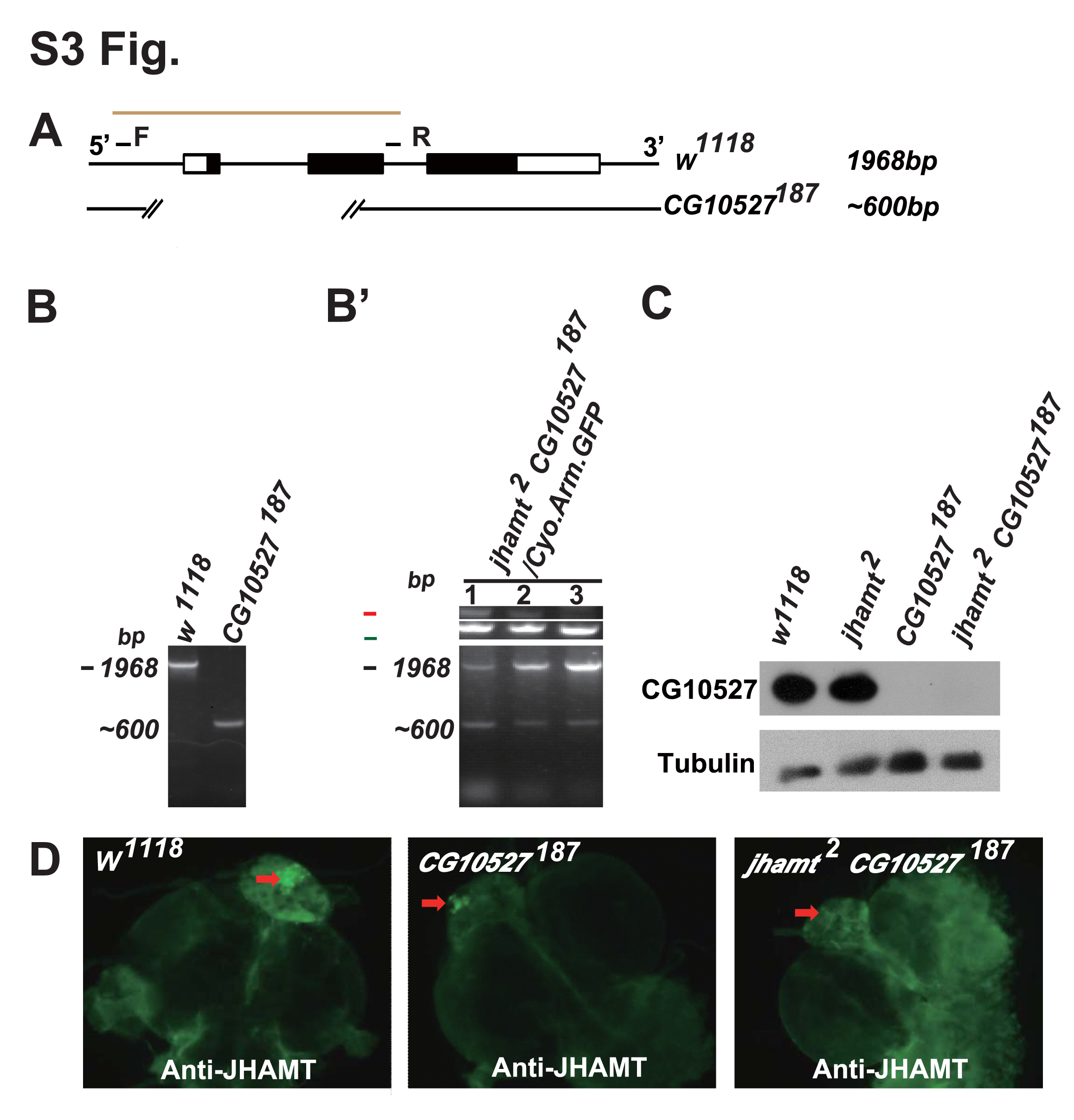

Supplement: S3 Fig — (A) Genomic structures of CG10527. CG10527 187 has an intragenic deletion of CG10527 compared to w 1118 [33]. The black boxes indicate the coding region, whereas the white boxes denote the non-coding exons. The black bars marked with F and R represent the primer pair CG10527-F/CG10527-R. The brown line indicates the PCR products (1968 bp length and ~600 bp length) obtained with the above primer pair using the genomic DNA extracted from w 1118 and CG10527 187 as templates, respectively. The blank region denotes the deletion region of CG10527 in CG10527 187. (B and B’) Three lines of the jhamt 2 CG10527 187 double mutants were confirmed by genomic DNA PCR. (B) The 1968-bp and ~600 bp PCR products were obtained with primer pair CG10527-F/CG10527-R (the black bars) from w 1118 and CG10527 187, respectively. (B’) The white PCR products of expected sizes with the primer pairs jhamt-1/jhamt-2 (the red bars) and jhamt-3/jhamt-4 (the green bars) (as shown in Fig. 1A) as well as the 1968 bp and ~600 bp PCR products with primer pair CG10527-F/CG10527-R (the black bars) were obtained in the 3 heterozygous jhamt 2 CG10527 187 lines (lane 1, 2 and 3). In the following experiments, the number 1 homozygous jhamt 2 CG10527 187 double mutant was used. (C) As detected by Western blot analysis, CG10527 was expressed in the brain-RG complexes of w 1118 and jhamt 2 but not those of CG10527 187 and jhamt 2 CG10527 187. Tubulin was used as the internal control. (D) Immunohistochemistry revealed no expression of JHAMT in the CA of jhamt 2 and jhamt 2 CG10527 187, while JHAMT was expressed in the CA of w 1118 and CG10527 187. Arrows indicate the CA. (TIF) [file pgen.1005038.s003.tif]

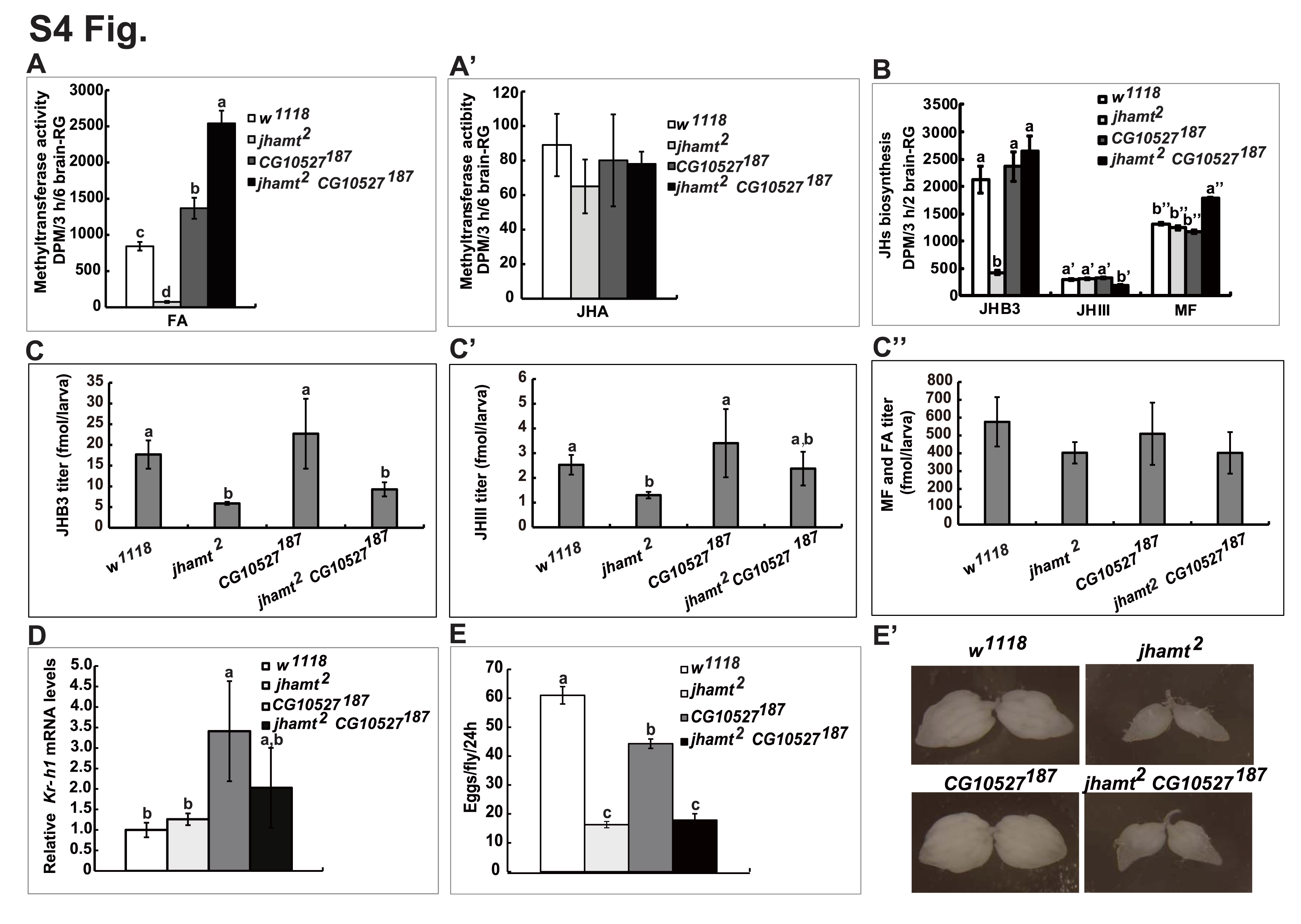

Supplement: S4 Fig — (A and A’) Measurements of methyltransferase activity of the brain-RG complexes in w 1118, jhamt 2, CG10527 187, and jhamt 2 CG10527 187 at 3h AIW using FA (A) or JHA (A’) as substrates. (B) Measurements of JH biosynthesis in the brain-RG complexes in w 1118, jhamt 2, CG10527 187, and jhamt 2 CG10527 187 at 3h AIW using the RCA-TLC method. (C-C”) Quantitative measurements of whole body titers of JHB3 (C), JH III (C’), and MF (C”) in w 1118, jhamt 2, CG10527 187, and jhamt 2 CG10527 187 at 3h AIW according to the HPLC-FD protocol. (D) qPCR measurements of the relative mRNA levels of Kr-h1 in the fat body tissues isolated from w 1118, jhamt 2, CG10527 187, and jhamt 2 CG10527 187 at 3h AIW. (E and E’) Comparisons of the average number of eggs laid by each pair of flies per 24 hours (E) and the ovary size of 6-day-old virgins (E’) among w 1118, jhamt 2, CG10527 187, and jhamt 2 CG10527 187. (TIF) [file pgen.1005038.s004.tif]

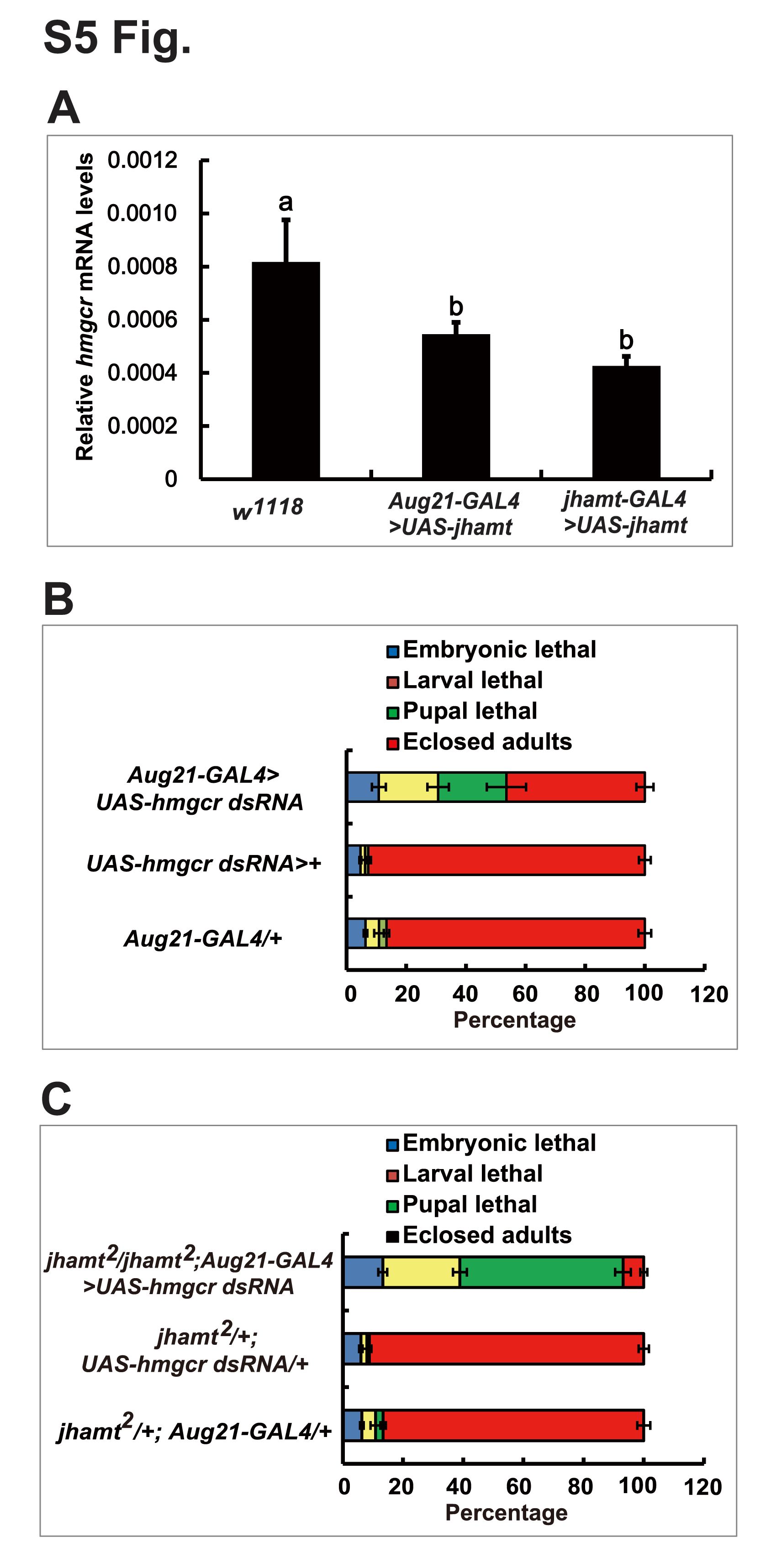

Supplement: S5 Fig — (A) RNAi efficiency of Aug-GAL4>UAS-hmgcr dsRNA and jhamt-GAL4>UAS-hmgcr dsRNA at 3h AIW. (B) Lethality of Aug21-GAL4>UAS-hmgcr dsRNA during the embryonic, larval, and pupal stages. Aug21-GAL4/+ and UAS-hmgcr dsRNA/+ were used as the controls. (C) Lethality of jhamt 2/jhamt 2; Aug21-GAL4>UAS-hmgcr dsRNA during the embryonic, larval, and pupal stages. jhamt 2/+; Aug21-GAL4/+ and jhamt 2 /+; UAS-hmgcr dsRNA/+ were used as the controls. (TIF) [file pgen.1005038.s005.tif]

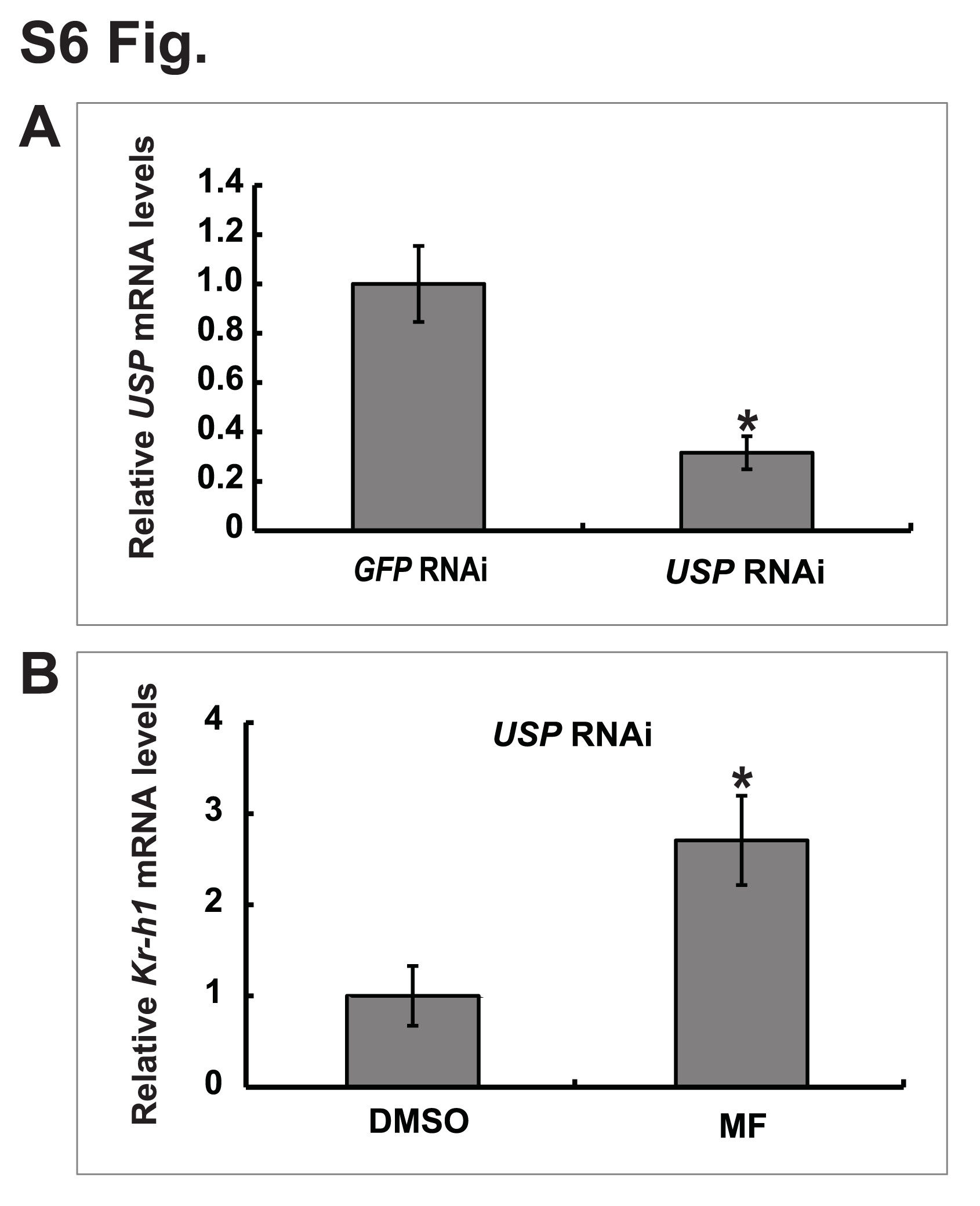

Supplement: S6 Fig — qPCR measurements of fold-changes of relative USP (A) and Kr-h1 (B) mRNA levels in Kc cells in which USP expression was reduced by RNAi (GFP RNAi and DMSO as a control) for 48 h, followed with treatments with MF (1×10^-10^~-6 M) for 30 min. (TIF) [file pgen.1005038.s006.tif]
