# Supplementary material for: Methyl Farnesoate Plays a Dual Role in Regulating Drosophila Metamorphosis
Source: PLoS Genet. 2015 Mar 16;11(3):e1005038. doi: 10.1371/journal.pgen.1005038 (PMC4361637; doi:10.1371/journal.pgen.1005038)
Supplement: S1 Table — (PDF) [file pgen.1005038.s007.pdf]

S1 Table. Primers used in this paper.

Primers	Sequences
jhamt-5'end-Not I	AGAATGCGGCCGCTAGGGGCTCATTATCCCA
jhamt-5'end-Acc65 I	CGGGGTACCGTTGGCGTGCTGATATAGAGA
jhamt-3'end-Asc I	GAATAGGCGCGCCGTTCCAACAATTGGTCTTAC
jhamt-3'end-BsiW I	GAATACGTACGGAAGCAGGACCTTCAATTGCG
jhamt-GAL4-SacII	CCGCGGCAACCCTTTGCTCTCAACATTTACG
jhamt-GAL4-BamH I	CCGCGGCAACCCTTTGCTCTCAACATTTACG
jhamt-1	GTGTATTTACTAGCTGTCATAGGCG
jhamt-2	TTGGTTGATTTCAGTAGTTGCAGTT
jhamt-3	TCTCTTATCTATCGCTACTTGGTTG
jhamt-4	CAATTATAATGCCGAAGCCTCTCTA
jhamt-5	CGGTAACGTGCTCATGGACT
jhamt-6	TTCGGGCTTCAGAAGATTGT
jhamt-7	TGACCATGTCACCTCGTTCTACTGC
jhamt-8	GAAGTCATCCAGGAACTGTTCATGC
CG10527-F	GAGGTGAAAGTCCAGGGAACAAA
CG10527-R	CCAACTCTACGAGTAGCGGGTA
Kr-h1-F	GAATACGACATAACAGCC
Kr-h1-R	CGATTTCCGTGAATATGTTCT
USP dsRNA-F	TAATACGACTCACTATAGGGAGAGAGTCCCGTGCCCTTCAT
USP dsRNA-R	TAATACGACTCACTATAGGGAGACCCTGGACACGCTATTGGT
USP-F	TTGGTCCCTATTCCACAGTCC
USP-R	GCGGCTTTCAGCAGAATCAC
hmgcr-F	CAGCGACATCTCCGAACT
hmgcr-R	ACGCACCACAGACATATCAA
